# Fukushima treated water release and marine sports

**DOI:** 10.3389/fpsyg.2026.1822678

**Published:** 2026-06-17

**Authors:** Jee-Hoon Han, Hye Ji Sa

**Affiliations:** 1 Department of Sport Culture, Dongguk University, Seoul, Republic of Korea; 2 Institute of Sports Science Convergence, Dongguk University, Seoul, Republic of Korea

**Keywords:** behavioral intention, Fukushima treated water, marine sports, perceived knowledge, psychological risk, theory of planned behavior

## Abstract

**Introduction:**

This study examined behavioral intentions toward marine sports participation among South Koreans in the context of the Fukushima Daiichi Nuclear Power Plant treated water discharge.

**Methods:**

Data were collected from 250 marine sports participants over 6 days beginning on September 6, 2023, and analyzed using SPSS and AMOS.

**Results:**

Attitudes toward marine sports did not significantly influence behavioral intentions, leading to the rejection of Hypothesis 1. In contrast, subjective norms and perceived behavioral control positively influenced behavioral intentions, supporting Hypotheses 2 and 3. Furthermore, subjective norms positively influenced attitudes, while perceived knowledge positively influenced both attitudes and subjective norms, supporting Hypotheses 4–6. In addition, perceived psychological risk negatively influenced attitudes and behavioral intentions, supporting Hypotheses 7 and 8.

**Discussion:**

These findings suggest that although attitudes alone may not directly determine behavioral intentions toward marine sports participation under environmental uncertainty, social influence, perceived control, perceived knowledge, and psychological risk play important roles in shaping behavioral responses. By incorporating perceived knowledge and psychological risk into the theory of planned behavior, this study provides additional insight into behavioral decision-making related to marine sports participation under environmental risk conditions.

## 1 Introduction

### 1.1 Research necessity and purpose

Treated radioactive water generated following the 2011 nuclear accident at the Fukushima Daiichi Nuclear Power Plant in Japan has been stored long-term. Starting on August 24, 2023, the Japanese government began discharging this water into the Pacific Ocean after processing it through the Advanced Liquid Processing System (ALPS) (Cabot, 2023). This decision triggered environmental, health, and safety concerns among Japan’s neighboring countries and the international community. Furthermore, the South Korean public has expressed increasing anxiety related to the marine environment around Fukushima. Despite the South Korean government’s efforts to alleviate these concerns, most South Koreans remain concerned about Japan releasing treated radioactive water into the ocean. Although Japan has maintained that the water is safe, opposition persists both domestically and internationally. Notably, China, Japan’s largest seafood trading partner, banned Japanese seafood imports in August 2023, although the ban was partially lifted in June 2025 (Associated Press, 2025; Yim, 2023). The Japanese government and the International Atomic Energy Agency (IAEA) have consistently emphasized that the treated water processed through the ALPS satisfies international safety standards. Nevertheless, despite these official explanations, public concern and distrust regarding the potential long-term environmental and health effects of the treated water discharge persist in neighboring countries, particularly South Korea (Chen et al., 2024). This discrepancy between official scientific assessments and public risk perception highlights the importance of understanding public psychological and behavioral responses toward marine-related leisure activities under environmental uncertainty.

Recent polls have shown that 80% of South Koreans are concerned about the release of treated water (Wong, 2023). Furthermore, many people, including fishers in the region, have expressed anxiety and anger regarding the Fukushima nuclear waste disposal plan, fearing the potential impact of the release of treated water on their livelihoods (Ng, 2023). The treated water poses direct risks to the Pacific region and will gradually spread throughout the central and northern Pacific, presenting potential hazards involving multiple factors (Liu et al., 2025).

One sector that could be directly affected by the psychological impact of treated water discharge is marine sports, which directly utilize the ocean. Marine sports are connected not only to leisure activities but also to marine tourism, the local economy, and marine culture. Thus, these interconnections underscore the pressing need for empirical research on the changing patterns of marine sports participation in response to the Fukushima treated water discharge.

Active sports tourism activities such as surfing, diving, and other water-based leisure activities depend significantly on the quality of aquatic environments and the condition of natural marine ecosystems. Previous research has suggested that environmental degradation and pollution directly influence tourists’ psychological responses and behavioral decisions, because these activities rely heavily on perceptions of safety and environmental sustainability (Kim et al., 2025). In particular, those who participate in marine sports tend to be highly sensitive to environmental threats, because the ocean constitutes the primary space for participation and experience. Furthermore, recent tourism studies have emphasized that nuclear and radiation-related environmental crises can significantly alter tourists’ perceptions, travel decisions, and protective behavioral responses, even when the risk is not directly observable (Chen et al., 2024). The release of Fukushima treated water, therefore, represents not only an environmental issue but also a tourism and leisure-related psychological crisis that can influence marine sports participation behaviors.

In South Korea, participation in marine sports has increased significantly, making marine sports a key component of the local leisure economy. However, the release of treated water from the Fukushima nuclear power plant not only poses an environmental risk but also represents a serious psychological barrier that could threaten this growing industry.

Despite the significant growth of marine sports participation and its central role in South Korea’s leisure economy, empirical research examining changes in the perceptions and behaviors of marine sports participants regarding the discharge of treated water from Fukushima is scarce. Most marine sports studies have focused on participation motives or environmental factors. Thus, the extraordinary crisis element of radiation and its impact on people’s marine activities have not been systematically analyzed. This study addresses this research gap by assessing how factors such as public psychological anxiety, information credibility, and risk perception influence the intention to participate in marine sports and actual participation behavior. Through this analysis, this study aims to improve public awareness of marine safety, establish crisis response strategies, and explore sustainable development directions for the marine sports industry.

This study first examines how marine sports participants’ perceptions of the discharge of treated water from Fukushima affect their attitudes, subjective norms, and perceived behavioral control. Second, it explains the intention to participate in marine sports using a model based on the theory of planned behavior (TPB), which has been expanded to include additional variables of perceived knowledge and perceived psychological risk. The TPB has previously been applied in tourism and leisure studies to identify various determinants of environmental behavior in eco-friendly actions (Ulker-Demirel and Ciftci, 2020; Yuriev et al., 2020).

### 1.2 Research hypotheses and model

**Figure 1:**
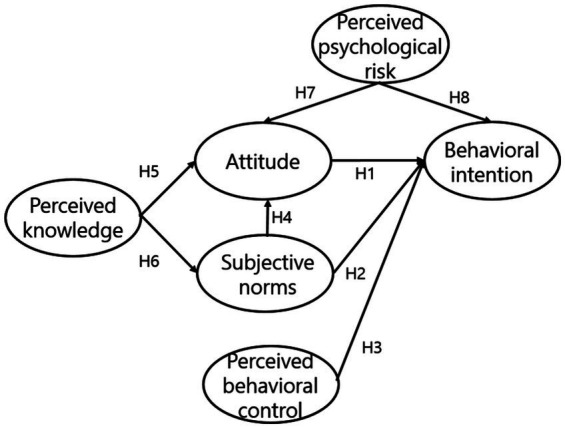
Research model.

Based on the theoretical framework of the TPB, hypotheses were formulated regarding attitudes, subjective norms, perceived behavioral control, perceived knowledge, perceived psychological risk, and behavioral intentions related to the release of treated water from Fukushima among marine sports participants. The research hypotheses are detailed below, and Figure 1 presents the research model.

Studies extending the TPB have found that attitudes, subjective norms, and perceived behavioral control significantly influence revisit intentions toward various tourist destinations (Soliman, 2021). Furthermore, research applying the extended TPB to examine eco-friendly restaurant usage has shown that these three factors positively influence visit intentions (Moon, 2021). Recent studies applying the TPB in sports and environmental risk contexts have consistently demonstrated that attitudes, subjective norms, and perceived behavioral control significantly predict behavioral intentions among sports participants. For example, Kim and Jeong (2024) reported that these TPB components significantly influenced outdoor sports participation intentions under perceived environmental risk conditions caused by particulate matter pollution. Their findings suggest that TPB remains a useful framework for explaining behavioral decision-making in sports participation contexts involving environmental uncertainty and perceived health threats.

According to the TPB, individuals who evaluate a behavior positively are more likely to form intentions to perform that behavior. In environmental risk situations, subjective norms become particularly influential because individuals tend to evaluate the safety and propriety of behaviors based on social expectations and the opinions of others. Furthermore, perceived behavioral control reflects individuals’ beliefs regarding their ability to safely and successfully perform a behavior despite external constraints or environmental uncertainties. Accordingly, attitudes, subjective norms, and perceived behavioral control are key determinants of behavioral intentions in contexts involving environmental risk and uncertainty (Ajzen, 1991; Han et al., 2020; Kim and Jeong, 2024).

*Hypothesis 1*: Attitudes toward marine sports positively influence behavioral intentions.

*Hypothesis 2*: Subjective norms regarding marine sports positively influence behavioral intentions.

*Hypothesis 3*: Perceived behavioral control in marine sports positively influences behavioral intentions.

Previous studies grounded in environmental psychology and the TPB have suggested that social norms and environmental knowledge significantly shape individuals’ evaluative attitudes and social perceptions regarding environmentally sensitive behaviors. In particular, Kim et al. (2025) demonstrated that perceived environmental knowledge positively influenced attitudes and subjective norms among active sports tourists participating in water-based leisure activities. In environmental risk situations, individuals tend to make evaluative judgments regarding participation behaviors based on socially shared information and collective expectations. Therefore, under environmental uncertainty caused by the Fukushima treated water release, subjective norms may substantially influence how individuals evaluate and form attitudes toward marine sports participation. Furthermore, prior studies have consistently reported that perceived knowledge enhances individuals’ environmental concern, attitudes, and pro-environmental behavioral tendencies by increasing awareness and understanding of environmental risks and sustainability issues (Han et al., 2020; Roh et al., 2022; Zeng et al., 2023). In particular, tourism studies applying the TPB have shown that perceived knowledge positively influences both attitudes and subjective norms when individuals evaluate safety-related behavioral decisions in uncertain environmental contexts (Han et al., 2020). Therefore, in the context of Fukushima treated water discharge, individuals with higher perceived knowledge may form stronger evaluative attitudes and become more sensitive to socially shared norms regarding marine sports participation. Based on these theoretical considerations, the following hypotheses are proposed:

*Hypothesis 4*: Subjective norms regarding marine sports positively influence attitudes.

*Hypothesis 5*: Perceived knowledge of marine sports positively influences attitudes.

*Hypothesis 6*: Perceived knowledge of marine sports positively influences subjective norms.

Previous studies have consistently reported that perceived psychological risk negatively influences attitudes and behavioral intentions in situations involving environmental uncertainty and health-related threats. In tourism and leisure contexts, individuals who perceive higher levels of environmental risk are more likely to form negative evaluations regarding participation behaviors, because perceived uncertainty and potential harm increase psychological discomfort and anxiety (Sánchez-Cañizares et al., 2021). Furthermore, recent studies examining nuclear and radiation-related tourism crises have suggested that invisible and difficult-to-control environmental threats can substantially influence individuals’ behavioral responses and protective decision-making processes (Chen et al., 2024). In the context of the Fukushima treated water discharge, individuals participating in marine sports may, therefore, perceive heightened psychological risk, because their activities involve directly interacting with the marine environment.

Previous research has also demonstrated that perceived risk significantly reduces behavioral intentions across various tourism and behavioral contexts by increasing uncertainty and reducing individuals’ willingness to engage in potentially threatening activities (Hamid and Bano, 2021). Accordingly, the following hypotheses are proposed:

*Hypothesis 7*: The perceived psychological risk of marine sports negatively influences attitudes.

*Hypothesis 8*: The perceived psychological risk of marine sports negatively influences behavioral intentions.

## 2 Theoretical background

### 2.1 Fukushima treated water and south Korean society

On the afternoon of March 11, 2011, a magnitude 9.0 earthquake and subsequent massive tsunami struck the coast of Miyagi Prefecture, Japan (Baba, 2013). The resulting accident at the Fukushima Daiichi Nuclear Power Plant released the largest number of radioactive nuclides into the terrestrial environment since the Chornobyl disaster in the former Soviet Union in 1986. The accident contaminated forests, farmland, grasslands, and urban areas. Therefore, long-term access to accident response data is necessary (Onda et al., 2020). Based on his study of marine sports experiences at the Fukushima site, Evers (2021) stated that everyone must now negotiate the sea as a toxic natural environment. Such experiences can engender new technologies, challenges, values, and orientations based on shifts in values, such as risk, health, and wellbeing. This highlights the need for a psychological assessment of marine sports from a South Korean perspective.

### 2.2 The extended TPB

Ajzen (1991) proposed the TPB as a social–psychological theory that predicts human behavior. According to the TPB, attitudes, subjective norms, and perceived behavioral control are antecedent variables that influence behavioral intentions, which subsequently lead to actual behavior. The TPB has been widely applied across various research fields. However, scholars have noted that the TPB has limitations in fully explaining individuals’ cognitive and emotional responses. Consequently, several studies have suggested extending the TPB by incorporating context-specific exogenous variables (Perugini and Bagozzi, 2001).

This study presents an extended TPB model designed for marine sports participants exposed to the environmental risk associated with the Fukushima treated water discharge. Specifically, this study examined behavioral intentions toward marine sports participation by incorporating perceived knowledge and perceived psychological risk alongside the original TPB variables.

Prior research has suggested that perceived knowledge is an important cognitive factor influencing attitudes, social perceptions, and behavioral decision-making (Boo and Park, 2013; Chan et al., 2014). Therefore, the extent to which marine sports participants believe they possess scientific or social knowledge regarding the treated water discharge may significantly influence their attitudes and subjective norms.

Perceived psychological risk refers to individuals’ feelings of anxiety, fear, or psychological discomfort regarding a specific situation (Olya and Han, 2020). In this study, perceived psychological risk was conceptualized as an emotional response to environmental uncertainty associated with the release of treated water from Fukushima rather than a direct negative evaluation of marine sports participation itself. The release of treated water may induce both environmental concern and psychological anxiety, which can negatively influence leisure and sports participation behaviors.

This extended TPB approach aims to more comprehensively explain how environmental uncertainty related to the Fukushima treated water discharge influences marine sports participants’ behavioral intentions. Although other contextual variables, such as trust and media influence, may also affect behavioral responses, this study specifically focused on perceived knowledge and perceived psychological risk because the Fukushima treated water issue simultaneously involves cognitive evaluation and emotional uncertainty regarding marine environmental safety.

## 3 Research method

### 3.1 Participants

Marine sports participants were selected for this study to examine their psychological behaviors regarding the release of treated water from Fukushima. Accordingly, the study population was defined as men and women who participate in marine sports. To ensure recent experience with the marine environment, the study sample consisted of individuals who had participated in marine sports such as surfing or diving at least once within the past year. Participants had engaged in various activities, including surfing, scuba diving, and sailing, ensuring diverse exposure to the marine environment. The sample size was determined using a structural equation modeling sample size calculator (Soper, 2021), and an online survey was conducted accordingly. Data were collected over 6 days, starting on September 6, 2023, with a specialized online survey agency collecting data from 250 individuals. The survey explained the research objectives and questionnaire content, and all participants had to provide consent before proceeding. Table 1 presents the participant characteristics. Because participation frequency and environmental exposure levels may vary depending on the type and intensity of marine sports activities, the findings may not generalize to all marine sport participants and should, therefore, be interpreted cautiously. In particular, some participants in the present study reported relatively low participation frequency, which may reflect broader public perceptions rather than the perspectives of highly committed marine sports participants.

**Table 1:** Participant characteristics.

Characteristic	Level	Frequency	Percentage
Gender	Male	152	60.8
	Female	98	39.2
Age	20s	49	19.6
	30s	68	27.2
	40s	63	25.2
	50s	70	28.0
Marine sports participation frequency	Once a year	58	23.2
	Twice a year	80	32.0
	Three times a year	39	15.6
	Four times a year	29	11.6
	Five or more times a year	44	17.6
Perceived danger of Fukushima treated water release	Yes	214	85.6
	No	36	14.4
Main type of marine sport participation	Snorkeling	83	33.2
	Scuba diving	18	1.8
	Open-water swimming	59	23.6
	Parasailing	1	0.4
	Surfing	21	8.4
	Water skiing/wakeboarding	19	7.6
	Rafting/canoeing/kayaking	22	8.8
	Banana boat/peanut boat	27	10.8
Total		250	100

### 3.2 Measurement tools

The extended TPB used in this study comprised attitudes, subjective norms, perceived behavioral control, perceived knowledge, perceived psychological risk, and behavioral intentions regarding marine sports in the context of the release of treated water from Fukushima. A total of 22 items were used. Seven items measured attitudes, and three items each measured subjective norms, perceived behavioral control, perceived knowledge, three psychological risks, and behavioral intentions (Ajzen, 1991; Han and Hyun, 2017; Han et al., 2020). All items were rated using a 5-point Likert scale. Participants’ general characteristics were examined across four items: gender, age group, frequency of participation in marine sports, and perceived risk regarding the discharge of treated water from Fukushima. All items were rated on a 5-point Likert scale, where 1 indicates “strongly disagree,” and 5 indicates “strongly agree.” Higher scores reflect a more positive attitude toward or stronger agreement with the concept.

### 3.3 Factor analysis and reliability verification

This study verified the validity, reliability, and fit of the measurement model. Confirmatory factor analysis (CFA) was conducted to assess model fit using the Tucker–Lewis index (TLI), comparative fit index (CFI), and root mean square error of approximation (RMSEA). Table 2 presents the CFA results. The TLI was 0.972, the CFI was 0.977, and the RMSEA was 0.058, indicating acceptable model fit according to the recommended criteria (Hu and Bentler 1999).

**Table 2:** Confirmatory factor and reliability analysis results.

Factor	Item	Estimate	Standard error	CR	AVE	*α*
Attitude	Participating in marine sports during the release of treated water from the Fukushima plant is not for me.	0.958	0.163	0.972	0.837	0.986
	1. A good thing					
	2. Desirable	0.957	0.160			
	3. Pleasant	0.957	0.188			
	4. Wise	0.947	0.186			
	5. Favorable	0.951	0.182			
	6. Enjoyable	0.949	0.225			
	7. Positive	0.959	0.189			
Subjective norm	In the context of the release of treated water from Fukushima, people who are important to me	0.937	0.208	0.911	0.774	0.948
	1. Think it’s okay for me to engage in marine sports					
	2. Support my engagement in marine sports	0.928	0.251			
	3. Understand that I participate in marine sports	0.917	0.296			
Perceived behavioral control	In the case of the release of treated water from Fukushima:	0.695	0.621	0.827	0.617	0.852
	1. Whether or not I engage in marine sports is entirely up to me					
	2. I can engage in marine sports whenever I want	0.877	0.313			
	3. I have the time, opportunity, and ability to participate in marine sports	0.867	0.308			
Perceived knowledge	1. I know more facts about the release of treated water from Fukushima than other people do	0.895	0.171	0.913	0.779	0.904
	2. I know more facts about the release of treated water from Fukushima than my friends do	0.909	0.157			
	3. I know more facts about the release than marine sports participants do	0.814	0.322			
Perceived psychological risk	Regarding the release of treated water from Fukushima:	0.919	0.220	0.933	0.823	0.952
	1. I am uncomfortable participating in marine sports					
	2. I experience unwanted anxiety while participating in marine sports	0.957	0.131			
	3. I feel unnecessary tension while participating in marine sports	0.923	0.212			
Behavioral intention	Regarding the release of treated water from Fukushima, I intend to participate in marine sports in the near future	0.958	0.121	0.927	0.809	0.952
	1. I intend to do so					
	2. I plan to do so	0.957	0.128			
	3. I will make an effort to do so	0.890	0.370			

*x*^2^ = 358.134; DF = 194; TLI = 0.972; CFI = 0.977; RMSEA = 0.058.

Reliability was assessed using Cronbach’s alpha coefficients, which ranged from 0.852 to 0.986, exceeding the recommended threshold of 0.70 (Van de Ven and Ferry, 1980). Convergent validity was evaluated using construct reliability (CR) and average variance extracted (AVE). The CR values ranged from 0.827 to 0.972, and the AVE values ranged from 0.617 to 0.837, exceeding the recommended thresholds of 0.70 and 0.50, respectively, thereby confirming satisfactory convergent validity (Bagozzi and Yi, 1988).

Discriminant validity was assessed using the Fornell–Larcker criterion. The square root of the AVE values ranged from 0.786 to 0.912 and exceeded the corresponding inter-construct correlation coefficients, supporting discriminant validity (Fornell and Larcker, 1981). In addition, the heterotrait–monotrait ratio (HTMT) was examined, and all HTMT values were below the recommended threshold of 1.0, further confirming discriminant validity (Henseler et al., 2015) (Table 3).

**Table 3:** HTMT analysis results.

Construct	1	2	3	4	5	6
1	–					
2	0.864	–				
3	0.424	0.484	–			
4	0.224	0.140	0.290	–		
5	0.511	0.547	0.309	0.050	–	
6	0.581	0.648	0.450	0.051	0.695	–

1: Attitude, 2: Subjective Norm, 3: Perceived Behavioral Control, 4: Perceived Knowledge, 5: Perceived Psychological Risk, 6: Behavioral Intention.

Finally, variance inflation factor (VIF) values were examined to assess multicollinearity among predictor variables. As shown in Table 4, all VIF values were below the recommended threshold of 5, indicating that multicollinearity was not a serious concern in the present study (Hair et al., 2019).

**Table 4:** Variance inflation factor (VIF).

Dependent variable	Independent variable	VIF
Attitude	Subjective norm	1.412
	Perceived knowledge	1.032
	Perceived psychological risk	1.391
Behavioral intention	Attitude	3,387
	Subjective norm	3,649
	Perceived behavioral control	1,241
	Perceived psychological risk	1,398

### 3.4 Data processing

The collected data were analyzed using SPSS WIN and AMOS. Frequency analysis, Cronbach’s alpha reliability analysis, correlation analysis, and CFA were conducted to examine the reliability and validity of the measurement model. Structural equation modeling and path analysis were subsequently performed to test the proposed hypotheses. In addition, because all variables were collected using self-reported questionnaires at a single point in time, Harman’s single-factor test was conducted to assess the potential influence of common method bias. The results indicated that the first factor accounted for less than 50% of the total variance, indicating that common method bias was not a serious concern in the present study.

## 4 Results

### 4.1 Correlation analysis

This study examined the correlations between the following variables among marine sports participants: attitudes, subjective norms, perceived behavioral control, perceived knowledge, perceived psychological risk, and behavioral intentions regarding marine sports in the context of the release of treated water from Fukushima. Table 5 presents the correlation analysis results. Subjective norms and attitudes showed the highest correlation (*r* = 0.836), whereas perceived knowledge and perceived psychological risk showed the lowest correlation (*r* = 0.036). Perceived psychological risk and behavioral intentions showed a negative correlation (*r* = −0.662).

**Table 5:** Correlation analysis.

Variable	*M*	SD	1	2	3	4	5	6
1	2.409	1.382	0.912					
2	2.542	1.275	0.836^**^	0.880				
3	3.500	0.990	0.388^**^	0.435^**^	0.786			
4	3.629	0.874	0.210^**^	0.129^*^	0.253^**^	0.883		
5	3.682	1.157	−0.494^**^	−0.520^**^	−0.279^**^	0.036	0.907	
6	2.781	1.202	0.563^**^	0.616^**^	0.406^**^	0.045	−0.662^**^	0.900

1: Attitude, 2: Subjective Norm, 3: Perceived Behavioral Control, 4: Perceived Knowledge, 5: Perceived Psychological Risk, 6: Behavioral Intention.

^*^p < 0.05, ^**^p < 0.01.

### 4.2 Model fit

Table 6 presents the results regarding fit for the structural model of attitudes, subjective norms, perceived behavioral control, perceived knowledge, perceived psychological risk, and behavioral intentions regarding marine sports in the context of the release of treated water from Fukushima. The model met the fit criteria, with a CFI of 0.962 (>0.90), TLI of 0.956 (>0.90), and RMSEA of 0.074 (<0.10).

**Table 6:** Model fit for the path analysis model.

*χ* ^2^	DF	CFI	TLI	RMSEA
465.565	198	0.962	0.956	0.074

### 4.3 Hypothesis testing

**Figure 2:**
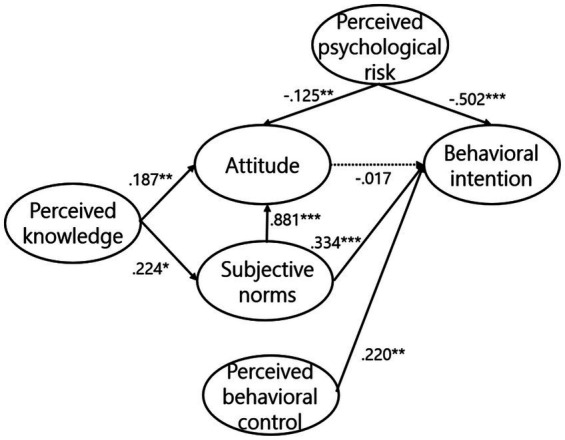
Path analysis results.

Table 7 and Figure 2 present the results of testing the hypotheses regarding the relationships among attitudes, subjective norms, perceived behavioral control, perceived knowledge, perceived psychological risk, and behavioral intentions toward marine sports in the context of the Fukushima treated water discharge.

**Table 7:** Path analysis results of the research model.

H	Path	Coefficient	S.E.	*t*	Status
H1	Attitude → Behavioral intention	−0.017	0.082	−0.213	Not supported
H2	Subjective norm → Behavioral intention	0.334	0.088	3.781^***^	Supported
H3	Perceived behavioral control → Behavioral intention	0.220	0.076	2.886^**^	Supported
H4	Subjective norm → Attitude	0.881	0.046	19.049^***^	Supported
H5	Perceived knowledge → Attitude	0.187	0.059	3.192^**^	Supported
H6	Perceived knowledge → Subjective norm	0.224	0.099	2.267^*^	Supported
H7	Perceived psychological risk → Attitude	−0.125	0.043	−2.935^**^	Supported
H8	Perceived psychological risk → Behavioral intention	−0.502	0.053	−9.459^***^	Supported

^***^p < 0.001, ^**^p < 0.01, ^*^p < 0.05.

The relationship between attitudes toward marine sports and behavioral intentions was not statistically significant. The path coefficient was −0.017, and the *t*-value was −0.213. Therefore, Hypothesis 1 was rejected.

Subjective norms regarding marine sports positively influenced behavioral intentions. The path coefficient was 0.334, and the *t*-value was 3.781, indicating statistical significance. Thus, Hypothesis 2 was supported (*p* < 0.001).

Perceived behavioral control in marine sports positively influenced behavioral intentions. The path coefficient was 0.220, and the *t*-value was 2.886, indicating statistical significance. Therefore, Hypothesis 3 was supported (*p* < 0.01).

Subjective norms regarding marine sports positively influenced attitudes, with a path coefficient of 0.881 and a *t*-value of 19.049, indicating statistical significance. Thus, Hypothesis 4 was supported (*p* < 0.001).

Perceived knowledge of marine sports positively influenced attitudes, with a path coefficient of 0.187 and a *t*-value of 3.192, indicating statistical significance. Thus, Hypothesis 5 was supported (*p* < 0.01).

Perceived knowledge of marine sports positively influenced subjective norms. The path coefficient was 0.224, and the *t*-value was 2.267, indicating statistical significance. Therefore, Hypothesis 6 was supported (*p* < 0.05).

Perceived psychological risk regarding marine sports negatively influenced attitudes, with a path coefficient of −0.125 and a *t*-value of −2.935. The negative relationship was statistically significant; therefore, Hypothesis 7 was supported (*p* < 0.01).

Finally, perceived psychological risk regarding marine sports negatively influenced behavioral intentions. The path coefficient was −0.502, and the *t*-value was −9.459, indicating statistical significance. Thus, Hypothesis 8 was supported (*p* < 0.001).

## 5 Discussion

This study conducted an empirical analysis by extending the TPB to examine the effects of the discharge of treated water from Fukushima on the behavioral intentions of marine sports participants. Testing the hypotheses yielded meaningful results and discussions.

Hypothesis 1, “attitudes toward marine sports will positively influence behavioral intentions,” was rejected because the results were not significant. This result suggests that in situations involving environmental problems, such as the discharge of treated water from Fukushima, individual attitudes toward marine sports may not directly influence behavioral intentions. Thus, external threats can weaken the relationship between attitude formation and behavioral intentions, as emphasized by previous studies. Quintal et al. (2010) used the TPB to explore the influence of risk and uncertainty on travel decision-making and found that attitudes toward visiting Australia did not influence behavioral intentions among South Korean outbound travelers. Furthermore, several studies have confirmed the lack of a consistently significant direct link between attitudes and behavior. For example, a study using European Union data revealed an attitude–behavior gap between the environmental and social domains. This finding demonstrates that social norms, constraints, and situational contexts can indirectly determine actual behavior (Borges-Tiago et al., 2024). Moreover, attitudes do not always directly predict behavioral intentions in the United Kingdom and Sweden, where cultural and group factors strongly influence behavior (Bock et al., 2010). Considering this context, the finding that the relationship between attitudes and behavioral intentions is not significant suggests that factors other than attitudes are crucial in behavioral decision-making.

The support for Hypothesis 2, “subjective norms regarding marine sports will positively influence behavioral intentions,” indicates that others’ opinions or social pressure can strongly influence the formation of intentions among marine sports participants. During environmental crises, individuals may be more sensitive to the expectations and norms of their surroundings. Researchers extending the TPB have found that subjective norms positively influence revisit intentions toward tourist destinations (Soliman, 2021). Furthermore, studies applying the extended TPB to eco-friendly restaurant usage have identified subjective norms as a crucial determinant of behavioral intentions (Moon, 2021). Therefore, the results of this study align with those of prior research, indicating that subjective norms positively influence behavioral intentions.

The support for Hypothesis 3, “perceived behavioral control in marine sports will positively influence behavioral intentions,” demonstrates that individuals’ confidence in their ability to act within their given environment is a crucial factor in determining their intention to participate in marine sports. In environmental crises, perceived behavioral control is linked to an individual’s belief that they can manage and cope with risks. Ahmed et al. (2021) applied the TPB and found that perceived behavioral control can positively influence young consumers’ intentions to purchase organic food. Furthermore, Sánchez-Cañizares et al. (2021) studied the impact of the perceived risk of contracting COVID-19 on travel intentions and found that, as expected, perceived behavioral control directly and positively influenced travel intentions.

The support for Hypothesis 4, “subjective norms regarding marine sports will positively influence attitudes,” underscores that others’ perceptions play a crucial role in shaping attitudes toward marine sports. Research within the context of organic food consumption has found that subjective norms significantly influence attitudes toward purchasing organic food (Al-Swidi et al., 2014). Park’s (2000) examination of the relationship between attitudes and subjective norms suggested that social attitudes are significantly related to subjective norms.

Hypothesis 5, “perceived knowledge of marine sports will positively influence attitudes,” was also supported, suggesting that the accuracy and reliability with which participants perceive information related to marine sports are key factors in the formation of positive attitudes. Research on organic food consumption integrating consumer value theory and the theory of reasoned action (Roh et al., 2022) has shown that perceived knowledge significantly influences attitudes. Perceived knowledge has been reported to increase the intensity and stability of attitudes and enhance the predictability of behavior (Wallace et al., 2020). This study’s results align with this theoretical context, suggesting that higher perceived knowledge leads individuals to perceive their attitudes as more firmly established and positive.

The results also supported Hypothesis 6, “perceived knowledge of marine sports will positively influence subjective norms,” indicating that individuals with sufficient knowledge are more likely to respond positively to social pressure or norms. Previous studies have shown that higher knowledge levels correlate with greater sensitivity to social expectations and norms. For example, using an Instagram campaign experiment, Varni et al. (2024) demonstrated that knowledge enhancement positively influences subjective norms and behavioral intentions. Tamar et al. (2021) revealed that knowledge positively influences subjective norms, attitudes, and perceived behavioral control, thereby promoting eco-friendly behavior. Therefore, this study’s finding that perceived knowledge positively influences subjective norms aligns with the findings of prior research, which have been repeatedly validated across various domains.

Hypothesis 7, “perceived psychological risk of marine sports will negatively influence attitudes,” was also supported. This suggests that higher perceived risks associated with participating in marine sports are more likely to lead to negative changes in attitudes toward participation. Research across various fields indicates that perceived risk negatively influences attitudes. Studies on mobile social networking services and online shopping have confirmed that higher psychological, social, and performance risks lead to more negative attitude changes (Kim and Kim, 2014). Furthermore, Sobkow et al. (2016) empirically demonstrated that negative emotions and stress increase risk perception, which in turn negatively changes attitudes toward the object. Moreover, research on the effects of the COVID-19 pandemic has demonstrated that high risk perception reinforces negative emotions and attitudes (Zhou et al., 2024). Therefore, the findings of this study align with broad empirical evidence and theoretical explanations, indicating that perceived risk negatively influences attitudes.

Finally, the findings strongly supported Hypothesis 8, “perceived psychological risk of marine sports will negatively influence behavioral intentions,” highlighting that the greater the perceived environmental or psychological risk, the more sharply the intention to engage in behavior may decrease. Perceived risk has been reported to have a negative effect on behavioral intentions across contexts. Research in diverse domains, including travel, consumer behavior, and health behavior, has repeatedly found that perceived psychological risk negatively influences behavioral intentions. For example, in a study on autonomous vehicle acceptance, Zhu and Jiao (2025) reported that risk perception reduces behavioral intentions. Similarly, Ma et al. (2025) investigated consumer behavior in overseas direct purchases and found that risk weakens trust and behavioral intentions.

Furthermore, research conducted during the COVID-19 pandemic found that risk perception directly influenced preventive behavioral intentions, such as wearing a mask (Lin et al., 2024). Within travel research, multiple studies have reported that risk perception strongly and negatively influences behavioral intentions (Nam and Yang, 2025). Therefore, the findings of this study reaffirm the tendency for risk perception to weaken not only attitudes but also behavioral intentions. Thus, strategies to mitigate risk perception are needed to promote behavior intention.

Collectively, these findings indicate that marine sports participants’ behavioral intentions are influenced more significantly by internal and external factors such as subjective norms, perceived behavioral control, and perceived psychological risk than by attitudes. Furthermore, perceived knowledge and psychological risk play crucial roles in marine sports participants’ attitudes and behavioral intentions. Higher perceived knowledge positively shaped attitudes and subjective norms, suggesting that acquiring sufficient information is essential for marine sports participants’ decision-making processes. Conversely, higher perceived psychological risk was more likely to negatively alter attitudes toward participation and behavioral intentions. Thus, psychological risk factors can inhibit behavioral intentions in environmental crises. Therefore, strategies that consider participants’ social norms and perceived risks are required to promote future participation in marine sports or respond to environmental crises.

Beyond hypothesis testing, this study demonstrated that the behavioral intentions of marine sports participants are more significantly influenced by subjective norms, perceived behavioral control, and perceived psychological risk than by attitudes. Specifically, behavioral intentions are strongly influenced by external factors, such as social context and risk perception, rather than personal preferences. Furthermore, in the South Korean context, the discharging of treated water from Fukushima is a particularly sensitive issue because of Fukushima’s geographical proximity and potential direct impact. This can significantly amplify the negative effect of risk perception on behavioral intentions and should be considered when interpreting this study’s results. Thus, South Korean marine sports participants are more sensitive to social pressure and environmental risk factors than to personal attitudes alone. Future East Asian regional studies or international comparative research should build on this important theoretical foundation.

## 6 Conclusion and recommendations

This study contributes to the literature by applying an extended TPB framework to the context of marine sports participation under environmental risk conditions associated with the release of treated water from Fukushima. Specifically, the findings suggest that subjective norms, perceived behavioral control, perceived knowledge, and perceived psychological risk play important roles in explaining behavioral intentions toward marine sports participation under environmental uncertainty. This study also provides additional insight into how environmental risk perception and socially shared concerns may influence behavioral decision-making in marine leisure contexts.

Furthermore, by incorporating perceived knowledge and psychological risk into the TPB framework, this study extends previous research examining behavioral intentions in tourism and leisure settings. Rather than focusing solely on traditional TPB variables, the findings highlight the importance of cognitive and emotional responses when individuals evaluate participation behaviors under perceived environmental threats. In particular, the results suggest that social influence and perceived psychological risk may affect behavioral intentions more significantly than individual attitudes in environmental crises.

From a practical perspective, this study suggests that the marine sports industry and policymakers should consider environmental crises when assessing participants’ perceptions and behavioral responses. Designing effective strategies that strengthen social norms and perceived behavioral control while mitigating psychological risk may help promote and sustain participation in marine sports. Policymakers and service providers should support the provision of accurate information, the implementation of risk management measures, and the strengthening of social communication systems to improve participants’ overall perceptions of marine safety. In particular, the findings highlight the importance of providing transparent and accessible information to reduce psychological uncertainty about marine environmental safety.

Demographic characteristics such as age and gender may influence environmental threat perceptions and behavioral intentions differently. Members of specific demographic groups may react more sensitively to environmental risks, indicating the need for more tailored approaches in future studies and practical strategies. In addition, environmental risk management and effective communication strategies in the marine sports sector may help improve participants’ perceptions of safety and intentions to continue participating in marine sports.

Despite its significant contributions, this study had several limitations. First, the sample was confined to citizens of South Korea, which may limit the generalizability of the findings. Future studies should, therefore, collect data from a range of countries and cultural contexts. Second, this study focused on specific TPB-related variables and may not fully capture the explanatory power of alternative theoretical frameworks. Third, the study relied on perceived knowledge, which reflects respondents’ subjective understanding rather than objective knowledge accuracy. Fourth, because the survey explicitly referenced the Fukushima treated water issue, the framing of the questionnaire may have influenced participants’ psychological responses and the strength of the observed relationships. In addition, although the results of Harman’s single-factor test suggested that common method bias was not a serious concern, the use of self-reported cross-sectional data may nonetheless be impacted by response bias. Future research should, therefore, adopt more diverse theoretical approaches and methodological designs to further examine behavioral intentions toward marine sports participation under environmental uncertainty.

## Data Availability

The raw data supporting the conclusions of this article will be made available by the authors, without undue reservation.
